# Supplementary material for: Observation of two types of charge-density-wave orders in superconducting La2-xSrxCuO4
Source: Nat Commun. 2019 Jul 22;10:3269. doi: 10.1038/s41467-019-11167-z (PMC6646325; doi:10.1038/s41467-019-11167-z)
Supplement: Supplementary file 1 — Supplementary Information [file 41467_2019_11167_MOESM1_ESM.pdf]

Supplementary Information

Observation of two types of charge density wave orders in superconducting $La_{2-x}Sr_xCuO_4$

Wen, Huang, Lee *et al.*

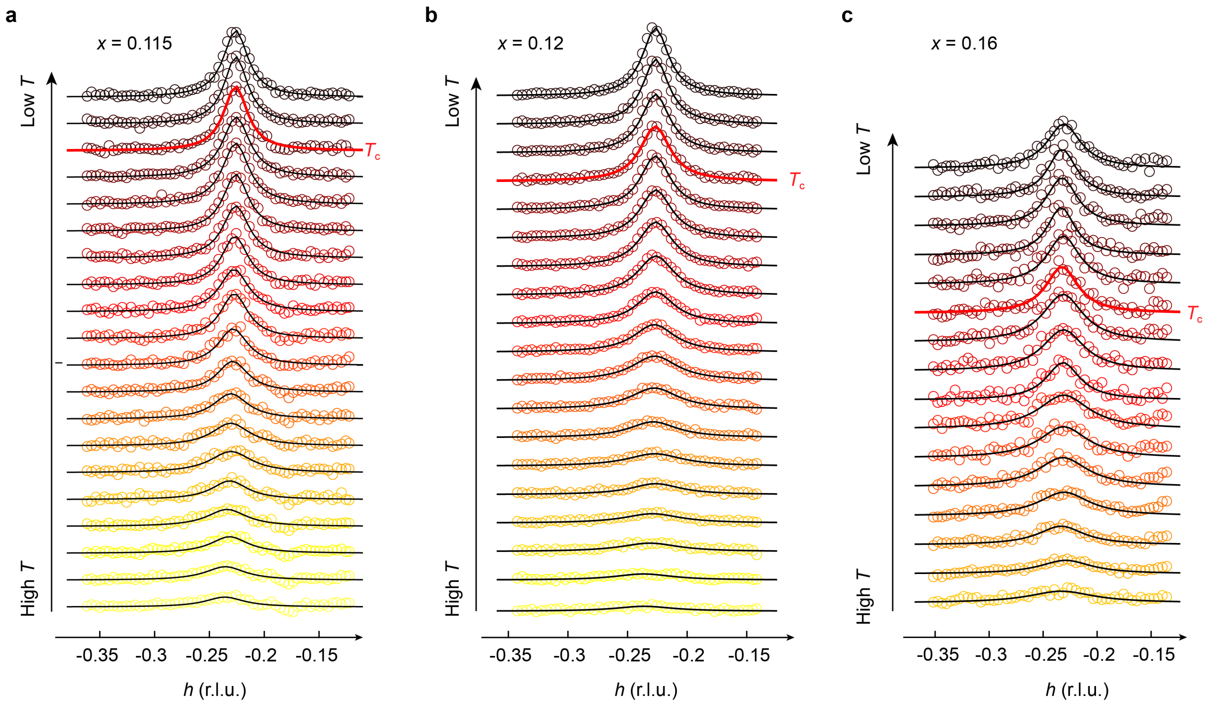

Supplementary Figure 1. Projected CDW profiles along h as a function of temperature for x = 0.115 (**a**), 0.12 (**b**) and 0.16 (**c**) LSCO. The solid lines are Lorentzian fits. The red solid lines correspond to the fits at respective T_c. As can be seen from this figure, the CDW intensities for the $x < x_{sdw}$ samples (i.e., x = 0.115 and 0.12) are found to increase even below T_c whereas in the x = 0.16 sample, the CDW intensity starts to decrease when cooling below T_c.

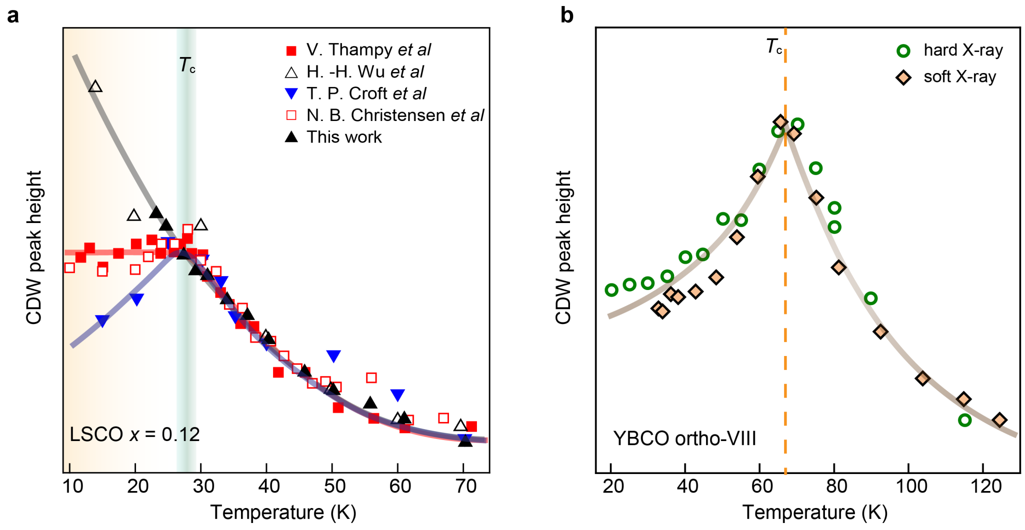

Supplementary Figure 2. **a** Temperature dependence of CDW peak height for $x = 0.12$ LSCO from all previously reported X-ray studies [1–4] as well as our result. For better comparison, the peak heights between different studies have been scaled. The vertical thick line denotes the temperature region around T_c. The solid lines are guides-to-the-eye. **b** Comparison of CDW behavior in ortho-VIII YBCO measured by hard X-ray scattering [5] and our soft X-ray scattering. The vertical dashed line denotes the T_c.

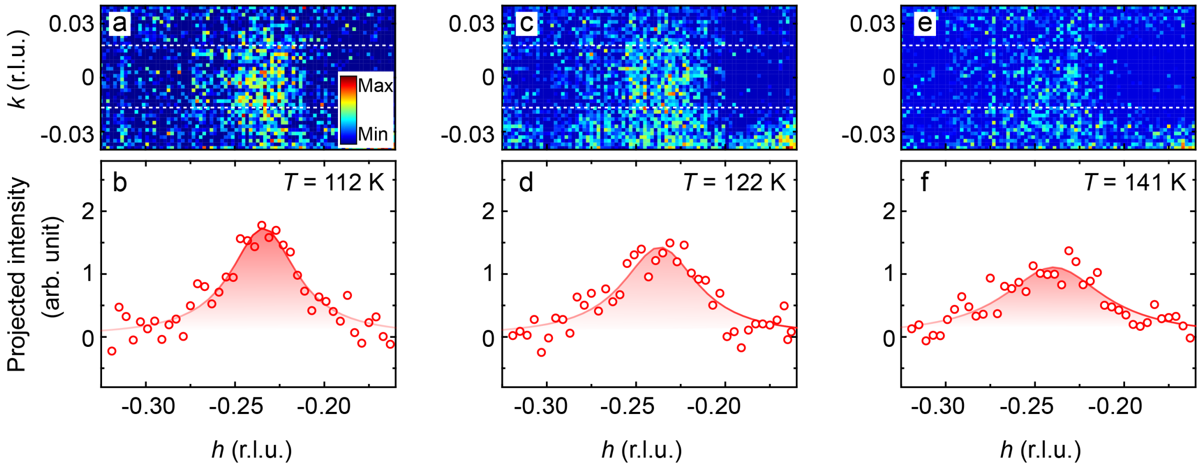

Supplementary Figure 3. CDW intensities for $x = 0.144$ below (112 K), near (122 K), and above (141 K) the pseudogap temperature $T^{*} \sim 120$ K. **a, c, e** show the scattering intensity maps on the *hk*-plane. **b, d, f** show the corresponding CDW peak after integrating the intensity map near the peak center between the two white dashed lines in the intensity maps. Solid curves are Lorentzian fits to the data. It clearly highlights that the CDW-SRO intensities persist above $T^{*} \sim 120$ K. In fact, a recent numerical study found evidence for stripe fluctuations at temperatures as high as ~ 1000 K [6]. As shown in the data at $T = 141$ K, the CDW signal is approaching the noise level of the instrument as the temperature gets higher. Although our novel RSXS approach provides a much-improved detection limit, its sensitivity is still insufficient for detailed study of the CDW-SRO at even higher temperatures.

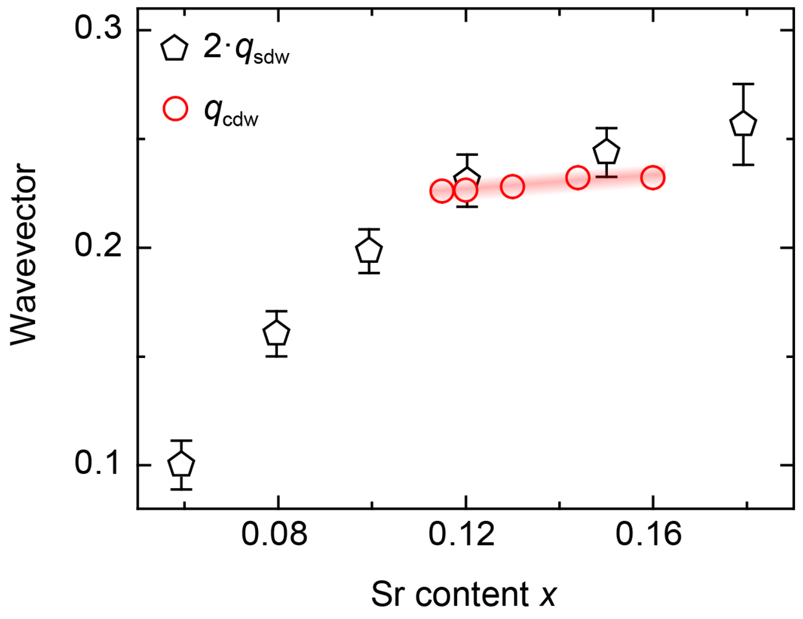

Supplementary Figure 4. Comparison of CDW and SDW ordering wave vectors in LSCO. The CDW wave vectors (q_{cdw}) are measured in this study, and the SDW wave vector (q_{sdw}) and its error are taken from ref. [7]. q_{cdw} for all 5 dopings of LSCO is estimated to be about 0.23 r.l.u. Note that q_{cdw} slightly changes with doping. As discussed in the main text, q_{cdw} and $2q_{\mathrm{sdw}}$ show a reasonable overlap in the doping range between $x = 0.11$ and 0.16, indicating mutual commensurability between CDW and SDW ($q_{\mathrm{cdw}} \sim 2q_{\mathrm{sdw}}$), which is expected for the stripe order. The error bar for q_{cdw} is smaller than the symbol size, which represents 1 standard deviation (s.d.) of the fit parameter.

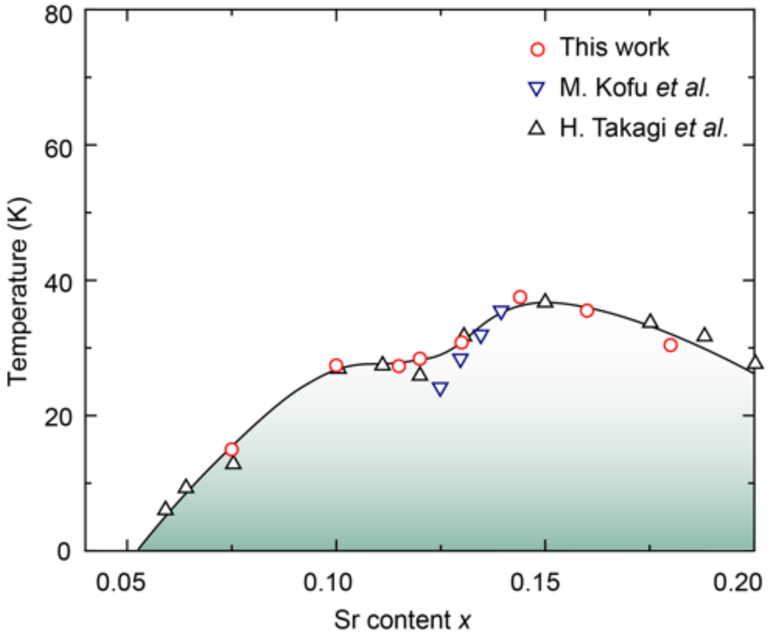

Supplementary Figure 5. The superconducting phase diagram of LSCO. T_c obtained from our measurements are in good agreement with previously reported data in ref. [8, 9]. The colored shade and line are guides-to-the-eye.

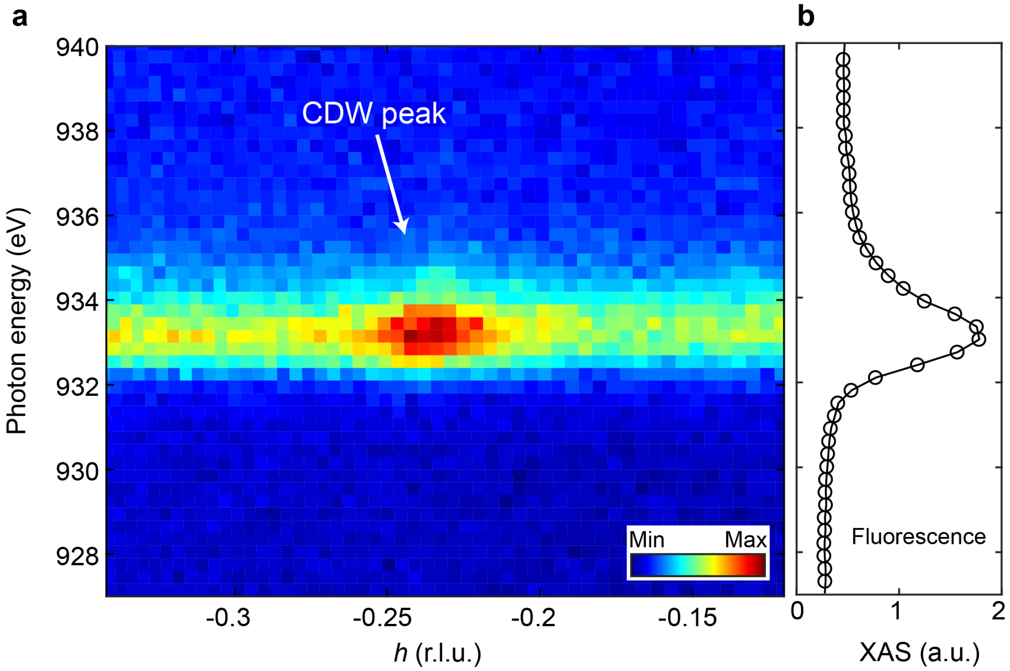

Supplementary Figure 6. **a** The energy resonant profile of the CDW peak for $x = 0.13$ LSCO measured at $T = 24$ K. **b** The corresponding X-ray absorption spectroscopy (XAS) near the Cu L_3 edge measured through the fluorescence yield. Since the CDW order is associated with Cu orbitals, there is a clear energy resonance around the photon energy ~ 933.3 eV, which is close to the Cu L_3 absorption maximum. As shown in the figure, a small residual fluorescence background remains around the Cu L_3-region in our RSXS approach, which is likely due to the inelastic scattering processes.

Supplementary Discussion

As described in the main text, the CDW in LSCO has been observed using both hard (non-resonant) and soft (resonant) X-ray scattering in the doping range $0.11 \leq x \leq 0.13$ [1–4]. Most of the reported works focused on the $x = 0.12$ LSCO since a strong CDW signal is expected for $x \sim 1/8$. There are subtle but important differences between our results and the earlier results.

Supplementary Fig. 2a shows a comparison of all the previously reported temperature dependence of the CDW peak height [1–4] to our measurements for the same doping of $x = 0.12$. The temperature-dependent behaviors above T_c are very similar. However, below T_c, there are apparent differences between different studies – the CDW peak height increases [2], decreases [1], or levels off [3, 4] upon cooling below T_c. Our results support the increasing trend. Note that although it is not shown here, the reported CDW correlation length in [1, 3, 4] keeps increasing below T_c, which is consistent with our result.

Our identification of two types of CDW orders in LSCO provides a natural explanation for the seemingly different experimental results for $x = 0.12$ LSCO. As we have shown in the main text, the low temperature state in $x = 0.12$ LSCO features a mixture of CDW stripe order and CDW-SRO. The CDW stripe order develops below T_c, while CDW-SRO is suppressed by superconductivity. Since both are simultaneously probed in the X-ray scattering measurements, whether the resultant total CDW intensity increases or decreases below T_c would depend on the relative volume fraction between the CDW stripe order and CDW-SRO that is probed in different measurements.

Supplementary Fig. 2b shows a comparison of the CDW intensities for ortho-VIII YBCO measured by hard X-ray and soft X-ray. The good agreement is consistent with our explanation above – since only CDW-SRO is present in the YBCO sample (SDW order is absent), similar CDW behavior is expected regardless of the probing volume in different measurements.

Supplementary References

1. Croft, T., Lester, C., Senn, M., Bombardi, A. & Hayden, S. Charge density wave fluctuations in $La_{2-x}Sr_xCuO_4$ and their competition with superconductivity. *Phys. Rev. B* **89**, 224513 (2014).6. H.-H. Wu *et al.*, *Nat. Commun.* **3**, 1023 (2012).

2. Wu, H.-H. *et al.* Charge stripe order near the surface of 12-percent doped $La_{2-x}Sr_xCuO_4$. *Nat. Commun.* **3**, 1023 (2012).

3. Thampy, V. *et al.* Rotated stripe order and its competition with superconductivity in $La_{1.88}Sr_{0.12}CuO_4$. *Phys. Rev. B* **90**, 100510 (2014).

4. Christensen, N. *et al.* Bulk charge stripe order competing with superconductivity in $La_{2-x}Sr_xCuO_4$ (x = 0.12). Preprint at https://arxiv.org/pdf/1404.3192 (2014).

5. Chang, J. *et al.* Direct observation of competition between superconductivity and charge density wave order in $YBa_2Cu_3O_{6.67}$. *Nat. Phys.* **8**, 871 (2012).

6. Huang, E. W. *et al.* Numerical evidence of fluctuating stripes in the normal state of high-T_c cuprate superconductors. *Science* **358**, 1161 (2017).

7. Yamada, K. *et al.* Doping dependence of the spatially modulated dynamical spin correlations and the superconducting-transition temperature in $La_{2-x}Sr_xCuO_4$. *Phys. Rev. B* **57**, 6165–6172 (1998).

8. Kofu, M., Kimura, H. & Hirota, K. Zn and Ni effects on the low-energy spin excitations in $La_{1.85}Sr_{0.15}CuO_4$. *Phys. Rev. B* **72**, 064502 (2005).

9. Takagi, H., Ido, T., Ishibashi, S. & Uota, S. Superconductor-to-nonsuperconductor transition in $(La_{1-x}Sr_x)_2CuO_4$ as investigated by transport and magnetic measurements. *Phys. Rev. B* **40**, 2254 (1989).
